# Comorbidities and co-medications in populations with and without chronic hepatitis C virus infection in Japan between 2015 and 2016

**DOI:** 10.1186/s12879-018-3148-z

**Published:** 2018-05-24

**Authors:** Daniel J. Ruzicka, Jumpei Tetsuka, Go Fujimoto, Tatsuya Kanto

**Affiliations:** 1 Medical Affairs, MSD K.K, Kitanomaru Square, 1-13-12 Kudan-kita, Chiyoda-ku, Tokyo 102-8667 Japan; 2 Biostatistics and Research Decision Sciences, MSD K.K, Kitanomaru Square, 1-13-12 Kudan-kita, Chiyoda-ku, Tokyo 102-8667 Japan; 3 The Research Center for Hepatitis and Immunology Department of Liver Disease, National Center for Global Health and Medicine, 1-7-1 Kohnodai, Ichikawa, Chiba 272-8516 Japan

**Keywords:** Chronic hepatitis C, Comorbidity, Drug interactions, Japan, Polypharmacy

## Abstract

**Background:**

Direct-acting anti-viral agents have improved the treatment of chronic hepatitis C virus (HCV) infection, but this treatment is challenging for patients using co-medications because of potential drug–drug interactions. This study aimed to examine the comorbidities and co-medications of Japanese chronic HCV patients by age group, compared with a non-HCV patient population.

**Methods:**

This was a retrospective observational study using a hospital-based medical claims database. We extracted data of patients with chronic HCV aged ≥18 years, and age-, sex-, and hospital-matched patients without HCV, for the period from January 2015 to November 2016, and then examined chronic comorbidities, long-term co-medications, and medications prescribed at least once during the study period.

**Results:**

We analysed data from 128,967 chronic HCV patients and 515,868 non-HCV patients. The median age was 70 years, and 51.0% of patients were male. More chronic HCV patients than non-HCV patients (70.5% vs. 47.1%) had at least one comorbidity, and older patients had more comorbidities than younger patients. The most common comorbidities in chronic HCV patients were diseases of oesophagus, stomach and duodenum (41.7%), followed by hypertensive diseases (31.4%). Chronic HCV patients used co-medications more commonly than non-HCV patients, and older patients used more co-medications. The most common long-term co-medications in chronic HCV patients were proton pump inhibitors (14.0%), which were prescribed to 31.9% of chronic HCV patients at least once during the study period.

**Conclusions:**

Patients with chronic HCV in Japan had more comorbidities than patients without chronic HCV regardless of age. Particularly older patients, who constitute the majority of the HCV patient population in Japan, commonly had multiple comorbidities and used co-medications. To optimise HCV treatment, physicians need to know the exact medication profiles of patients and take appropriate action to manage drug–drug interactions.

**Electronic supplementary material:**

The online version of this article (10.1186/s12879-018-3148-z) contains supplementary material, which is available to authorized users.

## Background

The advent of direct-acting anti-viral agents (DAAs) has dramatically improved the treatment of hepatitis C virus (HCV) infection. Treatment with these agents can achieve high sustained virologic response rates with a favourable tolerability and shortened treatment duration [1, 2]. However, DAA treatment is challenging for patients who use co-medications, especially drugs metabolised by cytochrome P450 CYP3A4, because of potential drug–drug interactions (DDIs) [3].

Chronic HCV infection causes persistent inflammation, which can result in the development of both liver diseases (e.g. cirrhosis, hepatocellular carcinoma) and extrahepatic diseases [4, 5]. Chronic HCV patients therefore tend to have various comorbidities. Some comorbidities such as HIV or hepatitis B virus (HBV) co-infection, obesity, and insulin resistance can contribute to the progression of liver fibrosis, which can lead to advanced liver diseases [6, 7]. Moreover, it has been reported that chronic HCV patients also have an increased risk of death from extrahepatic diseases such as circulatory diseases, renal diseases, and non-liver cancers compared with non-HCV patients [8]. Thus, management of these comorbidities is important to prevent disease progression and reduce mortality from both liver diseases and extrahepatic diseases. However, the use of co-medication to treat comorbidities, regardless of whether it is long- or short-term, can complicate HCV treatment because of the risk of DDIs with DAAs [3, 9]. The situation is even more challenging when treating older patients because they tend to have more comorbidities and use more co-medications [10].

Physicians therefore need to understand the comorbidities and co-medication profiles of chronic HCV patients to appropriately manage chronic HCV infection along with their comorbidities. In the US, studies have examined the comorbidities and co-medications of chronic HCV patients [9, 11]. However, to our knowledge, no such studies have been conducted in Japan, although the profile of chronic HCV patients there may differ from elsewhere. In Japan, the prevalence of HCV infection is higher in older people [12, 13]. HCV started to spread in the 1930s through intravenous drug abuse or medical procedures such as blood transfusion and the use of contaminated needles. However, transmission through blood transfusion has dramatically decreased over the past 50 years thanks to the discovery of HCV and the introduction of HCV antibody screening of donor blood [14], resulting in a low prevalence of HCV infection among younger people [15]. Because of the age distribution of the patient population and potential international differences in treatment patterns, it is important to obtain real-world data about comorbidities and co-medications in chronic HCV patients in Japan.

The aim of this study was therefore to describe the prevalence of comorbidities and the use of co-medications in chronic HCV patients in Japan by age group, compared with an age-, sex-, and hospital-matched non-HCV patient population, using a large-scale medical claims database. This study will create new data that are not yet available in Japan, which will provide physicians with the better understanding of chronic HCV patients in Japan.

## Methods

### Study design and data source

This was a retrospective, observational database study to examine the number and types of comorbidities and co-medications by age group in patients with and those without chronic HCV. Data were extracted from a hospital-based medical claims database in Japan, which was constructed by Medical Data Vision Co., Ltd. As of January 2017, the database contained data on over 17 million patients in 288 hospitals in Japan, all of which are hospitals with advanced medical care capabilities (e.g., acute care hospitals, general hospitals) and use the diagnosis procedure combination/per-diem payment system. Of these hospitals, 176 (61.1%) had 200–499 beds, 59 (20.5%) had fewer than 200 beds, and 53 (18.4%) had 500 beds or more. The database contained both inpatient and outpatient claims data from any department of the hospitals, including age, sex, diagnoses, medical procedures, prescriptions (inpatient/outpatient prescription, claim name and code, dose, prescription days), and hospitalisation details (if any). Because this hospital-based claims database was not linked to patients’ medical records, any medical and treatment histories recorded at different hospitals could not be traced.

### Study population

We extracted data on patients aged ≥18 years with at least one record of chronic HCV (the International Statistical Classification of Diseases and Related Health Problems 10th Revision [ICD-10] code: B18.2) between January 2015 and November 2016 (the study period). As non-HCV controls, we extracted data of patients who had no record of chronic HCV (B18.2) during the study period, who were matched to chronic HCV patients according to age (age group), sex, and hospital. The matching ratio of chronic HCV patients to non-HCV controls was 1:10. Patient age was calculated in the month of the first record during the study period, and patients were grouped into the following age ranges: 18–24, 25–29, 30–34, 35–39, 40–44, 45–49, 50–54, 55–59, 60–64, 65–69, 70–74, 75–79, 80–84, 85–89, and ≥ 90 years.

Chronic HCV patients who had prescription records of either DAAs or peginterferon plus ribavirin were defined as treated. The type of HCV treatment was defined as either DAAs or peginterferon plus ribavirin depending on the patients’ latest record of HCV treatment. Patients who had no record of DAAs or peginterferon plus ribavirin combination therapy during the study period were considered to be untreated.

### Definitions of comorbidities and co-medications

#### Comorbidities

A comorbidity was identified using ICD-10 codes and defined as another disease (i.e. excluding chronic HCV [B18.2] and chronic hepatitis [K73]) recorded over a time frame of 6 months or more during the study period. If the time from the first to the last record of a particular disease was 6 months or more, the disease was treated as a comorbidity. The minimum time frame of 6 months was set to target chronic diseases from a clinical viewpoint. Comorbidities were classified using the ICD-10 three-character code block categories (e.g. A00–A09 for intestinal infectious diseases) [16].

Among comorbidities, the ten relevant systemic diseases of interest were defined as follows: metabolic disorders (E70–E90); diabetes mellitus (E10–E14); cardiovascular diseases (ischaemic heart diseases [I20–I25], heart failure [I50], and cerebrovascular diseases [I60–I69]); hypertensive diseases (I10–I15); renal failure (N17–N19); dermatitis and eczema (L20–L30); diseases of oesophagus, stomach and duodenum (K20–K31); liver cancers (C22); neoplasms other than liver cancers (C00–D49 except for C22); and psychiatric disorders (mental and behavioural disorders [F00–F99] and sleep disorders [G47]).

#### Co-medications

Medications were classified using the fourth level of the EphMRA Anatomical classification system codes (ATC codes) [17]. A non-HCV-related medication was considered to be a co-medication if 1) it was supplied for a total of ≥180 days or 2) the ATC code was recorded in 6 or more consecutive months during the study period. DAAs, ribavirin, peginterferon, ursodeoxycholic acid, and monoammonium glycyrrhizinate/glycine/L-Cysteine hydrochloride hydrate were considered to be HCV-related medications, and not co-medications.

We targeted co-medications used for a long period. The total of 180 prescription days was a strict requirement because the study period was about 2 years. However, because patients need to visit the same hospital at least twice to receive medications to cover 180 days, we considered that the co-medications identified could probably be those used for the treatment of each patient’s chronic comorbid conditions.

Although we focused on long-term co-medications, we also examined the use of non-HCV-related medications prescribed at least once during the study period because even one-off or short-term use of a medication can influence the patient’s treatment regimen. As with co-medications, non-HCV-related medications prescribed at least once during the study period were classified using the fourth level of ATC codes. Examining medications used for a short period will shed light on the types of medications commonly used in Japanese chronic HCV patients, which might otherwise be underestimated using our strict definition of co-medications.

### Statistical analysis

The patients’ demographic and clinical characteristics were summarised descriptively for chronic HCV patients and non-HCV patients; these included age; sex; hospitalisation (the number of patients with hospital admission, the total length and number of hospital admissions); mental behavioural disorders due to use of alcohol (ICD-10 code F10), tobacco (F17), psychoactive substances (F12–16, F18–19), or opioids (F11); HIV co-infection (B20–24); and HBV co-infection (B18.0, B18.1). Disease names coded as chronic HCV (B18.2) and types of HCV treatment were summarised descriptively for chronic HCV patients.

Different types of comorbidities were summarised descriptively for each group. To compare the prevalence of different types of comorbidities in chronic HCV and non-HCV patients, the chi-square test was performed. The number of comorbidities by patient was summarised by age group for each patient group. Co-medications and non-HCV-related medications prescribed at least once during the study period were summarised descriptively by age group for each patient group, as was the number of co-medications by patient.

All statistical analyses were performed using SAS release 9.4 (SAS Institute Inc., Cary, NC, USA).

## Results

### Patient characteristics

We analysed data from 128,967 chronic HCV patients and 515,868 non-HCV patients (Table 1). The median age was 70 years and 70% of patients were aged between 60 and 84 years; 51.0% of patients were male. Patients with a record of hospital admission during the study period accounted for 43.0% of chronic HCV patients and 27.5% of non-HCV patients. In the chronic HCV group, 11,533 (8.9%) patients had HCV-related cirrhosis, and 17,244 (13.4%) patients were treated. Of these, 16,338 (94.7%) patients received DAAs and 906 (5.3%) received peginterferon plus ribavirin.

**Table 1:** Demographic characteristics of chronic HCV patients and non-HCV patients

	Chronic HCV patients (*n* = 128,967)		Non-HCV patients (*n* = 515,868)	
	n	(%)	n	(%)
Male	65,781	(51.0)	263,124	(51.0)
Age (years), median (range)	70	(18–107)	70	(18–109)
Age category (years)				
18–24	556	(0.4)	2224	(0.4)
25–29	779	(0.6)	3116	(0.6)
30–34	1388	(1.1)	5552	(1.1)
35–39	2095	(1.6)	8380	(1.6)
40–44	3252	(2.5)	13,008	(2.5)
45–49	4639	(3.6)	18,556	(3.6)
50–54	7008	(5.4)	28,032	(5.4)
55–59	9221	(7.1)	36,884	(7.1)
60–64	13,581	(10.5)	54,324	(10.5)
65–69	19,222	(14.9)	76,888	(14.9)
70–74	20,376	(15.8)	81,504	(15.8)
75–79	20,279	(15.7)	81,116	(15.7)
80–84	16,881	(13.1)	67,524	(13.1)
85–89	7525	(5.8)	30,100	(5.8)
≥ 90	2165	(1.7)	8660	(1.7)
Chronic HCV disease^a^				
Chronic hepatitis C	85,282	(66.1)	–	
Hepatitis C	42,802	(33.2)	–	
HCV-related cirrhosis	11,533	(8.9)	–	
Hepatitis C virus infection	2560	(2.0)	–	
Decompensated HCV-related cirrhosis	645	(0.5)	–	
Compensated HCV-related cirrhosis	434	(0.3)	–	
With treatment of HCV	17,244	(13.4)	–	
DAA	16,338	(94.7)	–	
Peginterferon + ribavirin	906	(5.3)	–	
Mental and behavioural disorders due to^b^				
Alcohol	515	(0.4)	1114	(0.2)
Tobacco	345	(0.3)	607	(0.1)
Psychoactive substance use	137	(0.1)	135	(0.0)
Opioid use	0	(0.0)	0	(0.0)
Co-infection^b^				
HIV	336	(0.3)	167	(0.0)
HBV	5368	(4.2)	4823	(0.9)
Hospital admission^c^	55,423	(43.0)	141,817	(27.5)
Total length of hospital admissions (days), median (range)	18	(1–678)	13	(1–684)
Total number of hospital admissions				
Once	32,492	(25.2)	100,483	(19.5)
Twice or more	22,931	(17.8)	41,334	(8.0)

Notes: HCV, hepatitis C virus; DAA, direct-acting antiviral agent; HBV, hepatitis B virus

^a^For diseases coded as chronic HCV (B18.2), the number and proportion of patients with that specific disease were shown

^b^For the following diseases, the number and proportion of patients with at least one disease record during the study period were shown: mental and behavioural disorders due to use of alcohol (F10); tobacco (F17); psychoactive substances (F12–16, F18–19); opioids (F11), HIV (B20–24), and HBV (B18.0, B18.1)

^c^All-cause hospital admissions during the study period were counted

### Comorbidities

**Fig. 1:**
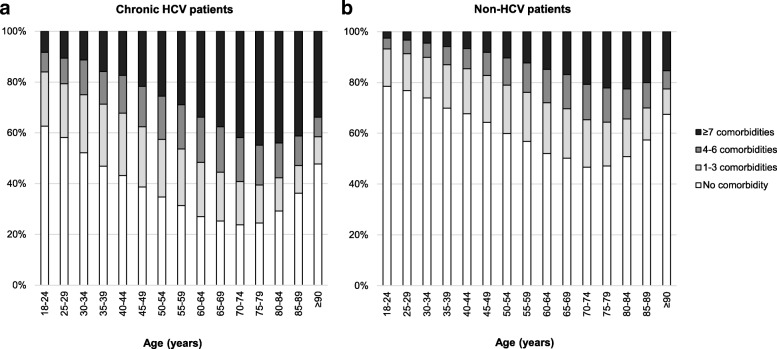
Proportions of patients with different numbers of comorbidities by age group. **a** chronic HCV patients and **b** non-HCV patients. Comorbidities were counted based on the ICD-10 three-character code block classification categories

Overall, 70.5% of chronic HCV patients and 47.1% of non-HCV patients had at least one comorbidity. Proportions of patients with 1–3, 4–6, and ≥ 7 comorbidities were 17.9, 15.9, and 36.7% in chronic HCV patients, and 17.6, 12.2, and 17.3% in non-HCV patients. Figure 1 shows the number of comorbidities by age group. In all age groups, the proportion of patients with at least one comorbidity was larger in chronic HCV patients than that in non-HCV patients. Patients in the older age groups tended to have more comorbidities than patients in the younger age groups, and 44.9% of chronic HCV patients aged 75–79 years had ≥7 comorbidities. In those aged 80 years or older, however, the proportions of patients with at least one comorbidity decreased.

The 20 most common comorbidities in chronic HCV patients are listed in Table 2. Diseases of oesophagus, stomach and duodenum were the most common (41.7%), followed by hypertensive diseases (31.4%) and metabolic disorders (28.2%). Aside from liver diseases, malignant neoplasms of digestive organs and gallbladder/biliary tract/pancreas disorders were much more prevalent in chronic HCV patients than in non-HCV patients (15.0% vs 4.6 and 11.2% vs 2.9%).

**Table 2:** The 20 most common comorbidities in chronic HCV patients and prevalence of relevant systemic diseases

		Chronic HCV patients (*n* = 128,967)		Non-HCV patients (*n* = 515,868)	
Type of comorbidities/relevant systemic diseases	ICD-10 code blocks	*n*	%	*n*	%
The 20 most common comorbidities					
Diseases of oesophagus, stomach and duodenum^♯^	K20-K31	53,817	41.7	94,325	18.3
Hypertensive diseases^♯^	I10-I15	40,466	31.4	79,602	15.4
Metabolic disorders^♯^	E70-E90	36,403	28.2	71,959	13.9
Diabetes mellitus^♯^	E10-E14	33,632	26.1	60,428	11.7
Other diseases of intestines	K55-K64	31,581	24.5	61,083	11.8
Episodic and paroxysmal disorders	G40-G47	25,077	19.4	44,756	8.7
Other dorsopathies	M50-M54	22,629	17.5	39,653	7.7
Diseases of liver	K70-K77	22,080	17.1	20,432	4.0
Malignant neoplasms of digestive organs	C15-C26	19,405	15.0	23,518	4.6
Other forms of heart disease	I30-I52	18,715	14.5	44,546	8.6
Dermatitis and eczema^♯^	L20-L30	16,294	12.6	24,107	4.7
Nutritional anaemias	D50-D53	15,433	12.0	20,678	4.0
Disorders of gallbladder, biliary tract and pancreas	K80-K87	14,471	11.2	14,977	2.9
General symptoms and signs	R50-R69	13,548	10.5	22,990	4.5
Ischaemic heart diseases	I20-I25	12,355	9.6	31,462	6.1
Chronic lower respiratory diseases	J40-J47	11,400	8.8	23,630	4.6
Disorders of bone density and structure	M80-M85	10,203	7.9	23,124	4.5
Other diseases of upper respiratory tract	J30-J39	10,168	7.9	20,404	4.0
Disorders of ocular muscles, binocular movement, accommodation and refraction	H49-H52	9941	7.7	30,291	5.9
Cerebrovascular diseases	I60-I69	9914	7.7	28,193	5.5
Relevant systemic diseases^a^					
Psychiatric disorders	–^b^	28,253	21.9	51,813	10.0
Neoplasms other than liver cancers	–^b^	26,286	20.4	79,126	15.3
Cardiovascular diseases	–^b^	24,000	18.6	62,901	12.2
Liver cancers	–^b^	11,878	9.2	1578	0.3
Renal failure	–^b^	5180	4.0	9293	1.8

*P*-values for the comparisons of the percentages in chronic HCV patients and non-HCV patients using the chi-square test were all < 0.0001

^a^Among the ten relevant systemic diseases of interest, five diseases that were not defined using ICD-10 three-character code block categories are listed under this heading. The other five diseases are listed in the ranking of the 20 most common comorbidities and indicated by a sharp (♯)

^b^These five relevant systemic diseases were defined as follows: psychiatric disorders included mental and behavioural disorders (F00–F99) and sleep disorders (G47). Neoplasms other than liver cancers were defined as C00–D49 except for C22. Cardiovascular diseases included ischaemic heart diseases (I20–I25), heart failure (I50), and cerebrovascular diseases (I60–I69). Liver cancers were defined as C22. Renal failure were defined as N17–N19

The prevalence of each type of relevant systemic diseases was higher in chronic HCV patients than that in non-HCV patients (Table 2). Neoplasms other than liver cancers were common in both groups, and present in 20.4% of chronic HCV patients and 15.3% of non-HCV patients. Only 4.0% of chronic HCV patients had renal failure.

**Fig. 2:**
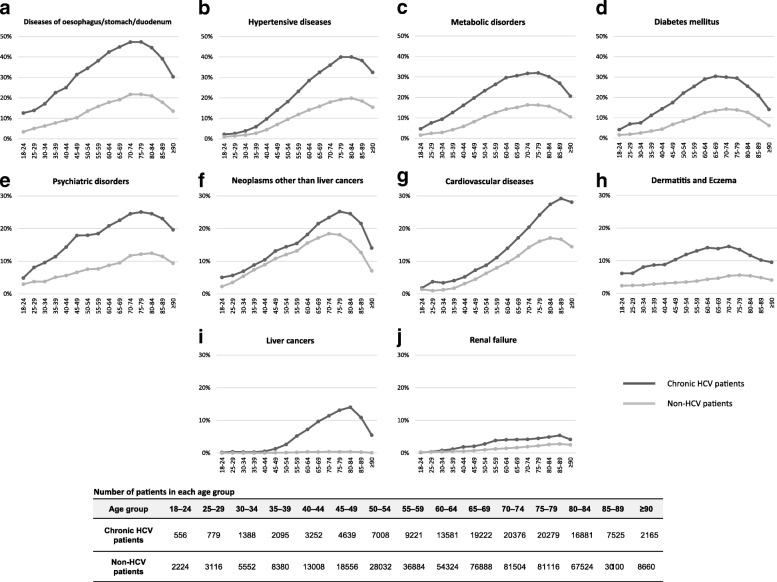
Prevalence of systemic diseases by age group in chronic HCV patients and non-HCV patients. **a** diseases of oesophagus, stomach and duodenum; **b** hypertensive diseases; **c** metabolic disorders; **d** diabetes mellitus; **e** psychiatric disorders; **f** neoplasms other than liver cancers; **g** cardiovascular diseases; **h** dermatitis and eczema; **i** liver cancers; and **j** renal failure

The prevalence of each systemic disease by age group is shown in Fig. 2. Diseases of oesophagus, stomach and duodenum were present in over 10% of chronic HCV patients, even in the youngest age group. Diabetes was more prevalent in chronic HCV patients aged between 60 and 79 years: about 30% vs. under 15% in non-HCV patients. Psychiatric disorders were common in chronic HCV patients aged 45 years or older, with a prevalence of approximately 20–25%. The prevalence of cardiovascular diseases was low in the younger age groups in both groups, but increased in older chronic HCV patients with a peak of 29.2% in the 85–89 year group. Although the prevalence of neoplasms other than liver cancers was similar in both groups in patients aged < 60 years, its prevalence exceeded 20% in chronic HCV patients aged 65–69 years and reached 25.2% in the 75–79 year group, in comparison to a peak prevalence of 18.4% in non-HCV patients aged 70–74 years.

### Co-medications

**Fig. 3:**
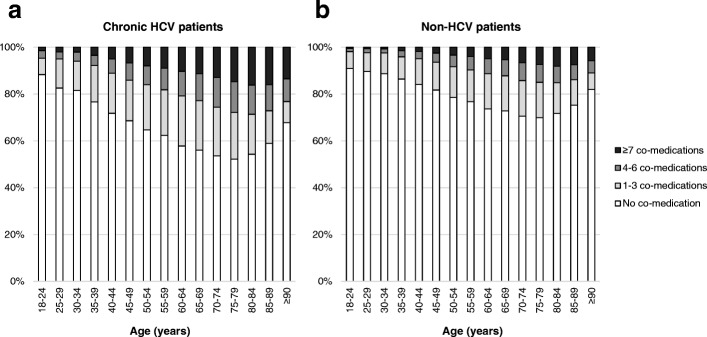
Proportions of patients with different numbers of co-medications by age group. **a** chronic HCV patients and **b** non-HCV patients

Overall, 41.9% of chronic HCV patients and 26.0% of non-HCV patients used at least one co-medication supplied for ≥180 days or recorded in at least 6 consecutive months. Of chronic HCV patients, 19.0% used 1–3 co-medications, 11.0% used 4–6, and 11.9% used ≥7 co-medications, compared with 13.8, 6.5, and 5.8% of non-HCV patients. Figure 3 shows the number of co-medications by age group. In all age groups, the proportion of patients with at least one co-medication was larger among chronic HCV patients. Patients in the older age groups tended to use more co-medications than patients in the younger age groups, and 16.2% of chronic HCV patients aged 80–84 years used ≥7 co-medications.

The most common co-medications in chronic HCV patients were proton pump inhibitors (14.0%), calcium antagonists (12.5%), and angiotensin-II antagonists (9.0%) (Table 3). Additional separate analysis of treated and non-treated chronic HCV patients revealed no major difference between these groups (Additional file 1: Table S1). Almost twice as many chronic HCV patients as non-HCV patients used these co-medications. In both groups, 5.8% of patients used statins as co-medications. Most common co-medications were used more by older patients. For example, proton pump inhibitors or calcium antagonists were used by over 15% of chronic HCV patients aged 75 years or over but by fewer than 5% of patients aged 18–44 years.

**Table 3:** Common co-medications and short-term non-HCV-related medications by age group

		Chronic HCV patients					Non-HCV patients				
Drug class	ATC Code	18–44 years	45–64 years	65–74 years	≥75 years	Total	18–44 years	45–64 years	65–74 years	≥75 years	Total
Number of patients		8070	34,449	39,598	46,850	128,967	32,280	137,796	158,392	187,400	515,868
Co-medications^a^											
Proton pump inhibitors	A02B2	4.8	11.8	15.1	16.1	14.0	1.7	4.6	6.6	7.1	6.0
Calcium antagonists, plain	C08A0	1.7	9.1	13.7	15.9	12.5	1.0	4.6	6.7	7.5	6.1
Angiotensin-II antagonists, plain	C09C0	2.0	7.7	9.9	10.4	9.0	1.2	4.2	5.5	5.4	4.8
Non-barbiturates, plain	N05B1	2.9	5.9	7.0	7.2	6.5	1.1	1.8	2.7	3.2	2.5
Plain antacids	A02A1	1.4	3.4	5.7	8.7	5.9	0.6	1.7	3.1	4.3	3.0
Statins (HMG-CoA reductase inhibitors)	C10A1	2.2	5.6	7.0	5.5	5.8	1.3	5.4	6.9	5.8	5.8
All other antiulcerants	A02B9	2.1	4.5	5.8	6.8	5.6	0.8	1.9	2.5	3.2	2.5
Loop diuretics plain	C03A2	1.0	3.8	5.1	7.8	5.5	0.2	1.1	1.6	2.9	1.9
DPP-4 inhibitor antidiabetics, plain	A10N1	1.5	5.2	6.0	4.8	5.1	0.6	2.8	3.4	2.7	2.8
Non-HCV-related medications prescribed at least once^b^											
Anaesthetics local, topical	N01B3	19.1	32.4	41.1	43.2	38.2	11.2	20.4	26.0	26.1	23.6
Non-narcotics and anti-pyretics	N02B0	24.9	26.5	31.2	38.6	32.2	20.8	16.5	18.2	22.2	19.4
Proton pump inhibitors	A02B2	15.8	27.1	32.5	37.7	31.9	8.0	14.5	17.7	20.9	17.4
Stimulant laxatives	A06A2	12.7	21.4	30.3	37.5	29.5	7.6	13.9	19.2	22.7	18.3
Anaesthetics local, medical injectables	N01B1	18.9	23.3	30.1	34.4	29.1	12.5	16.0	19.7	20.7	18.7
Plain antacids	A02A1	13.2	22.3	30.2	35.2	28.8	8.2	11.8	16.8	19.2	15.8
Anti-rheumatics, non-steroidal plain	M01A1	28.0	27.0	28.0	29.8	28.4	24.0	19.9	18.5	18.6	19.3
Plain antiflatulents and carminatives	A02A2	11.2	23.3	29.9	27.6	26.2	5.0	11.5	14.9	13.3	12.8
Injectable cephalosporins	J01D2	13.5	18.1	24.5	32.1	24.8	10.3	11.9	14.9	18.0	14.9
Calcium antagonists, plain	C08A0	4.6	16.3	25.0	33.7	24.6	2.7	9.2	13.7	18.5	13.5
Topical anti-rheumatics and analgesics	M02A0	9.8	16.9	22.7	29.2	22.7	6.2	10.5	14.1	18.2	14.1
Non-barbiturates, plain	N05B1	11.7	18.6	22.6	26.6	22.3	4.8	8.0	11.1	14.0	10.9
All other antiulcerants	A02B9	17.5	19.0	21.6	24.8	21.8	14.3	14.0	14.5	15.4	14.7

Data are percentages of patients in each age categories unless otherwise specified. For readability, patients were categorised into four age groups: 18–44, 45–64, 65–74, and ≥ 75 year groups

^a^Co-medications meet the requirements of either 1) supplied for a total of ≥180 days during the study period, or 2) the ATC codes were identified in 6 or more consecutive months during the study period. Co-medications prescribed in ≥5% of chronic HCV patients are listed

^b^Non-HCV-related medications prescribed at least once during the study period were examined. Medications prescribed in ≥20% of chronic HCV patients are listed. Classes of EphMRA ATC codes starting with K (hospital solutions) or T (diagnostic agents) were excluded from the list because these classes do not exist in WHO ATC classification and are difficult to interpret as “medications”: sodium chloride solutions (K01B1), low osmolar angio-urography (T01A0), 1/3 Electrolyte solutions (K01A4), and other electrolyte solutions (K01A9) are not shown in the table

In addition to co-medications, we also examined the non-HCV-related medications prescribed at least once during the study period to identify which medications are commonly used by chronic HCV patients. Table 3 shows the medications prescribed in ≥20% of chronic HCV patients (excluding medications classified as hospital solutions or diagnostic agents). Over 30% of chronic HCV patients aged ≥65 years used proton pump inhibitors or antacids, both of which were also common co-medications. These medications were used more commonly by chronic HCV patients compared with non-HCV patients (proton pump inhibitors, 31.9 and 17.4%; antacids, 28.8 and 15.8%, respectively). Anti-rheumatics were prescribed to approximately 28% of chronic HCV patients across all age groups.

## Discussion

To our knowledge, this is the first study to examine comorbidities and co-medications in chronic HCV patients in Japan. We found that chronic HCV patients had more comorbidities and used more co-medications than non-HCV patients. Older chronic HCV patients, who constitute the majority of the HCV patient population in Japan, commonly had multiple comorbidities and used more co-medications, suggesting the difficulty of appropriate management of this patient population.

In this study, older chronic HCV patients were more likely than younger patients to have comorbidities. Interestingly, however, the proportion of patients with comorbidities decreased in patients aged ≥75 years. This may be because those with fewer comorbidities tended to survive longer. It is also noteworthy that 16.0% of chronic HCV patients aged 18–24 years had four or more comorbidities, showing the relatively common presence of multiple comorbidities in young patients. Given that intravenous drug abuse is an important route of HCV transmission among young people [15], some of these young patients may be injecting drug users and are therefore more vulnerable to developing other diseases. However, these details could not be investigated in this study because of an absence of relevant data within the database.

The patterns of comorbidity distribution among chronic HCV patients were comparable to those of non-HCV patients who were 30 years older. The percentage of patients with no comorbidities decreased across the age groups in a very similar way in the two groups, although the proportion with no comorbidities was lower from a younger age in the chronic HCV group (see Fig. 1). A similar trend has been described for patients with ongoing HIV infection and it has been hypothesised that this might imply premature aging in those patients, possibly caused by persistent inflammatory and immune changes [18, 19]. Unfortunately, however, we did not examine whether similar changes occurred in chronic HCV patients.

The most common comorbidities in chronic HCV patients were diseases of oesophagus, stomach and duodenum (41.7%), hypertensive diseases (31.4%), and metabolic disorders (28.2%). A US study reported similar findings among chronic HCV patients: gastrointestinal disorders (24.4%), oesophageal disorders (20.5%), essential hypertension (32.6%), and disorders of lipid metabolism (25.9%) [11]. We also found that all ten systemic diseases were more common in chronic HCV patients than non-HCV patients, and that the prevalence of most systemic diseases tended to become higher in older chronic HCV patients. Our findings underscore the importance of age-related comorbidities such as hypertensive diseases, metabolic disorders, cardiovascular diseases and malignancies in older chronic HCV patients.

Chronic HCV patients used more co-medications than non-HCV patients, and more than one in five chronic HCV patients (22.9%) used at least four co-medications. Although direct comparisons of our results with previous findings are not appropriate because of different study methods, German studies have previously reported that 31–34% of HCV patients took at least four regular out-patient medications [3, 20]. An Italian study showed that 31.0% of chronic HCV patients with liver diseases used at least four co-medications, and estimated that 30–44% of chronic HCV patients receiving DAAs and using co-medications were at risk of DDIs [21]. The proportion in our study was slightly smaller than reported in these countries, but this may be because of our strict definition of co-medications. The common use of multiple co-medications in our study implies that chronic HCV patients in Japan may be at similarly high risk of potential DDIs.

The five most common co-medications used by chronic HCV patients were proton pump inhibitors, calcium antagonists, angiotensin-II antagonists, non-barbiturates, and antacids. This is similar to a German study reporting that the five most common co-medications included beta-blockers, proton pump inhibitors, thyroid hormones, angiotensin-II antagonists, and dihydropyridine calcium channel blockers [3]. The common use of gastric acid suppressants and hypertensive agents in our study might be attributed to our finding of a high prevalence of gastric diseases and hypertension in chronic HCV patients in Japan.

Gastric acid suppressants (e.g. proton pump inhibitors, antacids) have potential DDIs with DAAs [20–23]. These medications alter the acid environment, which can result in reduced absorption of certain drugs such as ledipasvir [22, 23]. Calcium antagonists also have potential DDIs with DAAs; plasma concentration of these medications can be increased by the concomitant use of CYP3A inhibitors such as telaprevir and simeprevir [22–24]. In this study, both these co-medications were common, especially in older chronic HCV patients. Although our chronic HCV population included a large proportion of untreated patients, examination of co-medication profiles in treated patients revealed that proton pump inhibitors and calcium antagonist were commonly prescribed to patients treated with DAAs (13.5 and 13.3%, respectively), roughly corresponding to the results of the overall chronic HCV population [see Table 3 & Additional file 1: Table S1]. Because of our strict definition of co-medications, our results probably reflect the medications used for chronic conditions, meaning that these medications may be difficult to stop or substitute. To select the optimal DAA treatment for chronic HCV patients, physicians should pay careful attention to the long-term use of these medications in their patients.

Even short-term use of other medications can influence DAA treatment. However, patients may not always inform their physicians about short-term use of medications, probably because they are not aware of the risks for DDIs. This study also examined short-term use of medications, and found that proton pump inhibitors, antacids, and calcium antagonists were used by about 25–30% of chronic HCV patients. Other classes of medications with potential DDIs with DAAs, such as analgesics, diuretics, and sedatives were also used by many chronic HCV patients [see Additional file 2: Table S2] [24, 25]. These results reinforce the need for physicians to have an exact knowledge of medication profiles of chronic HCV patients to avoid or manage DDIs. When initiating a DAA treatment, physicians may need to consider appropriate action for the management of DDIs, such as discontinuation, dose change, use of alternative medications, and increased monitoring of patients [23].

This study has several limitations. First, the generalisability of its results may be limited because the hospitals included in the database are only those with advanced medical care capabilities (e.g., acute care hospitals, general hospitals). The chronic HCV patients studied may therefore be “sicker” than other patients such as those treated at clinics. Likewise, the non-HCV patients may have severe medical conditions, which would influence the results. Second, the study has limitations inherent in the use of a pre-existing hospital claims database, such as misclassification owing to incorrectly recorded or missing data. For example, the diagnosis of chronic HCV infection was based on the diagnosis record and its accuracy might vary from hospital to hospital. Patients may have received other medications for other comorbidities at different hospitals, but this could not be traced. The lack of data on the time of HCV clearance in patients may have resulted in under- or overestimation of comorbidities. The low prevalence of HCV-related cirrhosis among chronic HCV patients may also be attributed in part to underestimation stemming from limitations of the database (i.e., miscoding, lack of data). However, this may also be explained by the difficulty of correct diagnosis of cirrhosis, which requires biopsy. Whereas patients usually undergo biopsy when they start HCV treatment, most of our chronic HCV patients were untreated, implying that not many patients who were likely to receive a correct diagnosis of HCV-related cirrhosis were included. Third, we set strict definitions to identify the chronic comorbidities and co-medications most likely to have been used to treat chronic conditions, which may have resulted in underestimation of both comorbidities and co-medications because patients may not constantly visit the hospital. Fourth, we examined ten relevant systemic diseases, but these are not comprehensive. Investigation of other extrahepatic manifestations is needed for a more comprehensive understanding of systemic manifestations in chronic HCV patients in Japan [see Additional file 3: Table S3 for additional data of additional extrahepatic manifestations of chronic HCV infection]. Finally, this study was unable to investigate any causal relationships between the results and chronic HCV infection or DAA treatment because the comorbidities and co-medications were not examined against the timing of diagnosis or treatment. Further studies may shed light on these relationships.

## Conclusions

In the present study in Japan, patients with chronic HCV had more comorbidities than those without HCV regardless of age. In particular, older chronic HCV patients commonly had multiple comorbidities and used more co-medications. The results suggest the difficulty of managing chronic HCV patients in Japan, especially older patients constituting the majority of the HCV patient population in Japan, because of the potential for DDIs. To provide optimal DAA treatment for this older patient population, it is important for physicians to closely monitor their medication profiles and take appropriate action to manage DDIs.

## Additional files

Additional file 1: **Table S1.** Common co-medications in treated and non-treated chronic HCV patients. Description of data: The proportions of patients who received common co-medications in treated chronic HCV patients (including patients treated with DAAs and patients treated with peginterferon plus ribavirin) and non-treated chronic HCV patients are summarised. (DOCX 17 kb)

Additional file 2: **Table S2.** Non-HCV-related medications prescribed at least once during the study period by age group. Description of data: The proportions of patients who received non-HCV-related medications at least once during the study period are summarised by age for both chronic HCV patients and non-HCV patients. (XLSX 184 kb)

Additional file 3: **Table S3.** Prevalence of other extrahepatic manifestations of chronic HCV infection. Description of data: Prevalence of additional extrahepatic manifestations that were not included in the analysis of 10 relevant systemic diseases in patients with or without chronic HCV. (DOCX 17 kb)

## Abbreviations

ATC codes: The EphMRA Anatomical classification system codes
DAA: Direct-acting anti-viral agent
DDI: Drug–drug interaction
HBV: Hepatitis B virus
HCV: Hepatitis C virus
ICD-10: The International Statistical Classification of Diseases and Related Health Problems 10th Revision
