# Supplementary material for: Comorbidities and co-medications in populations with and without chronic hepatitis C virus infection in Japan between 2015 and 2016
Source: BMC Infect Dis. 2018 May 24;18:237. doi: 10.1186/s12879-018-3148-z (PMC5968711; doi:10.1186/s12879-018-3148-z)
Supplement: Supplementary file 1 — Table S1. Common co-medications in treated and non-treated chronic HCV patients. Description of data: The proportions of patients who received common co-medications in treated chronic HCV patients (including patients treated with DAAs and patients treated with peginterferon plus ribavirin) and non-treated chronic HCV patients are summarised. (DOCX 17 kb) [file 12879_2018_3148_MOESM1_ESM.docx]

**Table S1. Common co-medications**^†^ **in treated and non-treated chronic HCV patients**

|  |  | Treated chronic HCV patients | | | | | |  | Non-treated chronic HCV patients | |
| --- | --- | --- | --- | --- | --- | --- | --- | --- | --- | --- |
|  |  | (n = 17,244) | | | | | |  | (n = 111,723) | |
|  |  | Total | | DAA  (n = 16,338) | | pegIFN + ribavirin  (n = 906) | |  |  | |
| Drug class | ATC Code | n | (%) | n | (%) | n | (%) |  | n | (%) |
| Proton pump inhibitors | A02B2 | 2,319 | (13.4) | 2,212 | (13.5) | 107 | (11.8) |  | 15,676 | (14.0) |
| Calcium antagonists, plain | C08A0 | 2,262 | (13.1) | 2,173 | (13.3) | 89 | (9.8) |  | 13,898 | (12.4) |
| Angiotensin-II antagonists, plain | C09C0 | 1,646 | (9.5) | 1,582 | (9.7) | 64 | (7.1) |  | 9,975 | (8.9) |
| Non-barbiturates, plain | N05B1 | 1,170 | (6.8) | 1,090 | (6.7) | 80 | (8.8) |  | 7,275 | (6.5) |
| All other antiulcerants | A02B9 | 1,008 | (5.8) | 959 | (5.9) | 49 | (5.4) |  | 6,192 | (5.5) |

Notes: HCV, hepatitis C virus; DAA, direct-acting antiviral agent

^†^Co-medications meet the requirements of either 1) supplied for a total of ≥180 days during the study period, or 2) the ATC codes were identified in 6 or more consecutive months during the study period. Co-medications prescribed in ≥5% of treated chronic HCV patients are listed.
