# Supplementary material for: Comorbidities and co-medications in populations with and without chronic hepatitis C virus infection in Japan between 2015 and 2016
Source: BMC Infect Dis. 2018 May 24;18:237. doi: 10.1186/s12879-018-3148-z (PMC5968711; doi:10.1186/s12879-018-3148-z)
Supplement: Supplementary file 3 — Table S3. Prevalence of other extrahepatic manifestations of chronic HCV infection. Description of data: Prevalence of additional extrahepatic manifestations that were not included in the analysis of 10 relevant systemic diseases in patients with or without chronic HCV. (DOCX 17 kb) [file 12879_2018_3148_MOESM3_ESM.docx]

**Table S3. Prevalence of other extrahepatic manifestations of chronic HCV infection**

| Diseases | ICD-10 code | Chronic HCV patients  (n=128,967) | |  | Non-HCV patients  (n=515,868) | |
| --- | --- | --- | --- | --- | --- | --- |
|  |  | n | (%) |  | n | (%) |
| Parkinson disease | G20 | 1,440 | (1.1) |  | 5,644 | (1.1) |
| Non-Hodgkin lymphoma | B21.2,  C82‒85, C91.5 | 2,100 | (1.6) |  | 4,502 | (0.9) |
| Cryoglobulinaemia | D89.1 | 399 | (0.3) |  | 60 | (0.0) |
| Monoclonal gammopathy of undetermined significance (MGUS) | D47.2 | 108 | (0.1) |  | 182 | (0.0) |
| Sicca syndrome | M35.0 | 1,047 | (0.8) |  | 2,632 | (0.5) |
| Inflammatory polyarthropathies | M05‒14 | 8,680 | (6.7) |  | 21,211 | (4.1) |

For each disease, the number and percentage of patients with at least one disease record during the study period are shown.
